# DArT Markers Effectively Target Gene Space in the Rye Genome

**DOI:** 10.3389/fpls.2016.01600

**Published:** 2016-10-26

**Authors:** Piotr Gawroński, Magdalena Pawełkowicz, Katarzyna Tofil, Grzegorz Uszyński, Saida Sharifova, Shivaksh Ahluwalia, Mirosław Tyrka, Maria Wędzony, Andrzej Kilian, Hanna Bolibok-Brągoszewska

**Affiliations:** ^1^Department of Plant Genetics, Breeding, and Biotechnology, Warsaw University of Life Sciences – SGGWWarsaw, Poland; ^2^Diversity Arrays Technology P/LBruce, ACT, Australia; ^3^Department of Biotechnology, Genetic Resources Institute of Azerbaijan National Academy of SciencesBaku, Azerbaijan; ^4^Kusuma School of Biological Sciences, Indian Institute of TechnologyNew Delhi, India; ^5^Department of Biotechnology and Bioinformatics, Rzeszow University of TechnologyRzeszow, Poland; ^6^Department of Genetics and Cytology, Pedagogical University of CracowCracow, Poland

**Keywords:** DArT, rye, *Secale*, functional annotation, genomics, *Triticeae* Pooideae

## Abstract

Large genome size and complexity hamper considerably the genomics research in relevant species. Rye (*Secale cereale* L.) has one of the largest genomes among cereal crops and repetitive sequences account for over 90% of its length. Diversity Arrays Technology is a high-throughput genotyping method, in which a preferential sampling of gene-rich regions is achieved through the use of methylation sensitive restriction enzymes. We obtained sequences of 6,177 rye DArT markers and following a redundancy analysis assembled them into 3,737 non-redundant sequences, which were then used in homology searches against five *Pooideae* sequence sets. In total 515 DArT sequences could be incorporated into publicly available rye genome zippers providing a starting point for the integration of DArT- and transcript-based genomics resources in rye. Using Blast2Go pipeline we attributed putative gene functions to 1101 (29.4%) of the non-redundant DArT marker sequences, including 132 sequences with putative disease resistance-related functions, which were found to be preferentially located in the 4RL and 6RL chromosomes. Comparative analysis based on the DArT sequences revealed obvious inconsistencies between two recently published high density consensus maps of rye. Furthermore we demonstrated that DArT marker sequences can be a source of SSR polymorphisms. Obtained data demonstrate that DArT markers effectively target gene space in the large, complex, and repetitive rye genome. Through the annotation of putative gene functions and the alignment of DArT sequences relative to reference genomes we obtained information, that will complement the results of the studies, where DArT genotyping was deployed, by simplifying the gene ontology and microcolinearity based identification of candidate genes.

## Introduction

Rye (*Secale cereale* L., 2n = 2x = 14, 1 C = 7917 Mbp; Dolezel et al., 1998) is a member of the *Poaceae* (grass) family, which comprises 12 subfamilies with 11,000 species in total, including other cereal crops and the model plant *Brachypodium* (Kellogg, 2015). Rye is a close relative of wheat and barley. These three species are members of the *Triticeae* tribe and shared a common ancestor ca. 13 million years ago (Gaut, 2002). Both wheat and barley were domesticated much earlier (ca. 10,000 BC; Fuller, 2007; Purugganan and Fuller, 2009) than rye, which occurred initially as a weed in fields of the other cereals. The intentional cultivation of rye began in Europe during the first millennium B.C. (Pre-Roman Iron Age; Behre, 1992; Fuller, 2007). Nowadays rye has high regional importance in Eastern, Northern, and Central Europe. In 2014, it was grown on 5.256 M hectares worldwide and 88.8% of the harvested rye grain was produced in Europe (FAOSTAT, 2016). Rye is mostly used for human consumption (bread making), animal feed, and industrial purposes (including alcohol production; Weipert, 2012). Rye grain contains high levels of dietary fiber (up to twice more than wheat) and several types of bioactive components, such as alkylresorcinols, sterols, phenolic acids, folates, and tocols. Therefore, rye wholegrain products can be considered healthy, functional food (Shewry et al., 2010; Koistinen and Hanhineva, 2015). Furthermore rye exhibits the highest cold/freezing tolerance among small-grain cereals, as well as considerable tolerance of other abiotic and biotic stresses, such as acidity, low soil fertility, and water deficiencies and it has been postulated that rye could thus constitute a model for functional analysis and future improvement of related cereals (Martis et al., 2013). However, little is known about the genes underlying these unique features of rye. The NCBI Gene database contains solely 111 sequences of *S. cereale* chloroplast genes (Middleton et al., 2014), in comparison, queries ‘*Triticum aestivum’* and ‘*Hordeum vulgare*’ produce 2218 and 617 results, respectively (accessed 18 May 2016). Therefore, there is an urgent need to intensify efforts that would extend the knowledge of the rye genome structure, speed up the establishment of association between sequence data and the targeted traits, and simplify candidate gene identification and map based gene cloning/isolation in rye.

Various processes, such as whole genome duplications (polyploidization events) followed by differential diploidization, aneuploidy, segmental duplications, chromosome breakage, and fusions, gain, and loss of repeat sequences (especially retrotransposon proliferation) influenced the structure of grass genomes during their divergent evolution (Gaut, 2002; Paterson et al., 2003; Bolot et al., 2009). In result grass species vary considerably with respect to genome size and basic chromosome number (Gaut, 2002). Nevertheless, as shown by comparative mapping studies, an extensive collinearity of grass genomes has been retained, permitting for the use of reference whole genome sequences of related model grasses in research on species, in which genome analysis is less advanced, e.g., in large genome cereals barley, wheat, and rye (Hackauf et al., 2009; Brenchley et al., 2012; Fluch et al., 2012). The *Triticeae* cereals are characterized by very large and complex genomes, with abundance of gene fragments and pseudogenes, and a very high proportion of repeated sequences -92% in the case of rye (Flavell et al., 1974). These features have hampered considerably genome sequencing in *Triticeae* crops (The International Wheat Genome Sequencing Consortium (IWGSC), 2014; Muñoz-Amatriaín et al., 2015) and for rye a reference genome sequence is still not available.

Recently, significant advances have been made in rye genomics. The first high-throughput genotyping technology was established for rye in 2009 with the development of a 1520-clone DArT genotyping panel (Bolibok-Bragoszewska et al., 2009). Since then, it was successfully applied for high density genetic map construction, both in rye and triticale (Bolibok-Bragoszewska et al., 2009; Milczarski et al., 2011; Tyrka et al., 2011, 2015), genome-wide germplasm characterization (Bolibok-Bragoszewska et al., 2014), QTL/gene mapping (Stojałowski et al., 2011; Miedaner et al., 2012; Myśków et al., 2012; Milczarski et al., 2016), and genomic selection (Wang et al., 2014, 2015; Schulthess et al., 2015). Following transcriptome sequencing a Rye5K SNP genotyping panel was developed and used for construction of high density transcript map (Haseneyer et al., 2011; Martis et al., 2013). Subsequently, low coverage chromosomal survey sequencing of flow sorted rye chromosomes was performed, and the resulting reads together with transcriptome assemblies were incorporated into rye genome zippers, which present a virtual linear gene order for seven rye chromosomes (Martis et al., 2013).

Diversity Arrays Technology is a high-throughput genotyping method, which does not rely on the availability of sequence information (Jaccoud et al., 2001). DArT markers are genomic fragments obtained in a genome complexity reduction procedure, which involves the use of a methylation sensitive endonuclease, usually *Pst*I. Therefore, DArT markers originate predominantly from the hypomethylated, low-copy, gene-rich genome regions. These genomic fragments are then cloned into pCR2.1-TOPO vector and introduced into *E. coli.* Individual colonies are picked into 384-well plates to produce a diversity panel. Inserts from the clones included in the diversity panel are then amplified, printed on microarrays, and used in genotyping (Xia et al., 2005). Sequence information for DArT markers of interest can be thus easily obtained through sequencing of amplified inserts. As demonstrated by several studies, this sequence knowledge can in turn can provide basis for the attribution of functional meaning to those markers (Tinker et al., 2009; Marone et al., 2012; Petroli et al., 2012; Aitken et al., 2014), and for candidate gene identification.

The aim of this study was to obtain and analyze sequence information for selected rye DArT markers. In particular we wanted to assess the efficiency of the DArT method in targeting low-copy regions in the very large, complex, and repetitive rye genome and to attribute functional meaning to DArT sequences in order to simplify future marker-trait associations and candidate gene identification. Furthermore, we (i) incorporated the newly obtained sequences into rye genome zippers providing a starting point for the integration of DArT- and transcript-based genomics resources in rye, (ii) mapped the rye DArT marker sequences *in silico* on the reference *Pooideae* genomes, (iii) demonstrated that DArT markers sequences can be a useful source of SSR markers.

## Materials and Methods

### DArT Clone Sequencing and Redundancy Analysis

For sequencing we selected DArT clones which fulfilled at least one of the following criteria: They were genetically mapped (Milczarski et al., 2011), they differentiated diverse rye accessions (Bolibok-Bragoszewska et al., 2014), they were located in the vicinity of QTLs for powdery mildew or leaf rust (Ciszkowicz et al., in preparation), or they were addressed to BAC clones following DArT based BAC library screening (Bolibok-Bragoszewska et al., 2015). The inserts of selected clones were amplified using the primers: ‘M13r nested improved’ (5’-TCACGACGTTGTAAAACGAC-3’) and ‘PCR2.1 fwd’ (5’-CAGGAAACAGCTATGACCATGATT-3’) and sent for sequencing by commercial Sanger sequencing service providers. The obtained sequence reads from both directions were merged into one sequence per clone where possible. Vector contaminations were removed based on results of a BLAST search against the GenBank UniVec database. *Pst*I adaptor (exclusive *Pst*I site) and low quality sequences were also trimmed. Additionally 50 sequences of wheat DArT markers included in the rye genotyping array, available through the Diversity Arrays website^1^, and 109 unpublished rye DArT marker sequences (available from A. Kilian upon request) were included in the analyses. DArT marker sequences were subsequently analyzed with the sequence assembly program CAP3 (Huang and Madan, 1999) using the default settings to identify singletons and sets (bins) of redundant sequences, arrange the latter into contigs, and to obtain consensus sequences. The resulting set of unique, non-redundant sequences (composed of singletons and consensus sequences) was then used in a BLAST search against the TREP database^2^ (Wicker et al., 2002) to identify DArT markers containing repetitive DNA sequences and in all subsequent analyses.

### Functional Annotation

For functional annotation of DArT sequences we performed BLASTX search against the non-redundant GenBank protein sequence database with default Blast2GO settings (Conesa et al., 2005; Conesa and Gotz, 2008). Annotation and mapping of gene ontology (GO) terms was done with the Blast2GO pipeline (Blast2GO v3.2). A more general GOSlim classification was obtained after using the GOSlim properties from Balst2GO data. The conversion to the raw GOSlim classification was performed according to the TAIR database (Berardini et al., 2004).

### Homology Search within *Pooideae* Genome Sequences and Rye Sequence Resources, Integration into Rye Genome Zippers

Genome sequences and annotation files of three *Pooideae* species: *Brachypodium distachyon* (Brachypodium_distachyon.v1.0.31.dna.toplevel) wheat [*T. aestivum*] (Triticum_aestivum.IWGSC1+popseq.31.dna.toplevel) and barley [*H. vulgare*] (Hordeum_vulgare.ASM32608v1.31.dna.toplevel) were downloaded from Ensembl Plants^3^. Sequences from Roche/454 shotgun sequencing of flow sorted individual rye chromosomes (sc454reads_anchored_all) – chromosomal survey sequences (CSS; Martis et al., 2013) and 115,400 contigs from rye transcriptome sequencing (Sce_Assembly03; Haseneyer et al., 2011) were downloaded from http://pgsb.helmholtz-muenchen.de/plant/rye/gz/download/ and http://www.gabipd.org/download/cgi-bin/Download.pl.cgi, respectively. A BLASTN homology search, with the *E*-value threshold of 10^-5^ was used to align the unique DArT sequences to the five *Pooideae* sequence sets mentioned above. Based on the study of Martis et al. (2013) we used different sequence identity settings for different sets: 75% for *Brachypodium*, 85% for barley, and 90% for wheat, Sce_Assembly03 and CSS. For all five sets two consecutive searches were done: with the minimum alignment length of 100 and 200 bp. Best hits against Sce_Assembly03 and CSS were then used to incorporate rye DArT markers sequences into Rye Genome Zippers (Martis et al., 2013) to generate a link between the DArT-based and transcriptome-sequencing-based genomics resources in rye.

### Sequence Based Comparison of Rye Consensus Maps

First, BioMercator V3 software (Sosnowski et al., 2012) was used to produce a composite genetic map based on the rye consensus map data from five RIL populations (Milczarski et al., 2011) and to obtain a linear order of DArT markers for each chromosome. Then, the positions of DArT markers in the composite map were compared with the position of the respective DArT markers sequences in the Rye Genome Zippers (Martis et al., 2013). The results were visualized using Circos (Krzywinski et al., 2009).

### Development of SSR Markers from Rye DArT Marker Sequences

Microsatellite motifs within DArT marker sequences were identified using MISA script (Thiel et al., 2003) with default settings. Primers for amplification of identified motifs were designed with Primer3 (Untergasser et al., 2012). A subset of primers was selected for experimental validation. In the validation experiments we used eight rye accessions: Two inbred lines, three landraces, two Polish population varieties, and one *S. vavilovii* accession. Each accession was represented by a pooled DNA sample obtained from several plants. Amplification, visualization and scoring of SSR markers as well as PIC value calculations were done as previously described (Targońska et al., 2016).

## Results

### DArT Marker Sequencing and Redundancy Analysis

In total 6,018 DArT marker sequences were obtained in this study, with the cumulative length of 3,048,961 bp and an average sequence length of 507 bp. The sequences have been submitted to the GenBank GSS database to be released upon publication (accession numbers KS366081 through KS372098, library accession number LIBGSS_039286). The final sequence set used in further analyses, which additionally included 50 wheat DArT markers sequences from the Diversity Arrays Technology web site and 109 unpublished rye DArT marker sequences, consisted of 6,177 DArT marker sequences (corresponding to 53.6% of the rye genotyping panel), with the cumulative length of 3,140,801 bp (508 bp on average). The complete list of DArT markers, which sequences were used in this study is given in the **Supplementary Table S1**.

**FIGURE 1:**
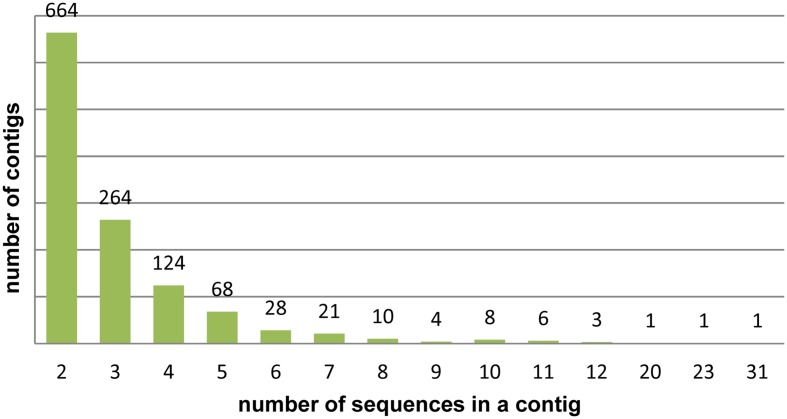
**Assembly of rye DArT sequences**. Number of sequence contigs of different sizes.

Following CAP3 analysis 3,643 DArT sequences were assembled into 1,203 contigs, the remaining 2,534 sequences were singletons (redundancy 39.5%). The number of sequences per contig varied from 2 to 31, the majority of the contigs (77%) contained two or three sequences (**Figure 1**). The resulting set of 3,737 unique, non-redundant sequences was used in subsequent analyses. A list of unique DArT sequences is given in the **Supplementary Table S2**. The consensus sequences were named after the longest sequence in the contig, with the suffix ‘_con’.

**FIGURE 2:**
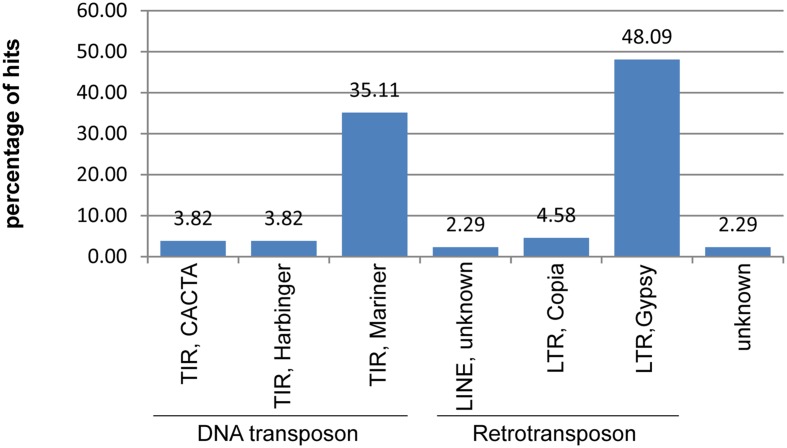
**Repetitive content of the non-redundant rye DArT marker sequences**. Relative frequencies of different classes of transposable elements identified within DArT markers sequences.

A search against the TREP database revealed that 78 of unique DArT markers (1.26% of the analyzed set) contained sequences displaying homology to the total of 46 repetitive elements. Frequency of BLAST hits to various repeat types is shown in **Figure 2**.

### Functional Annotation

**FIGURE 3:**
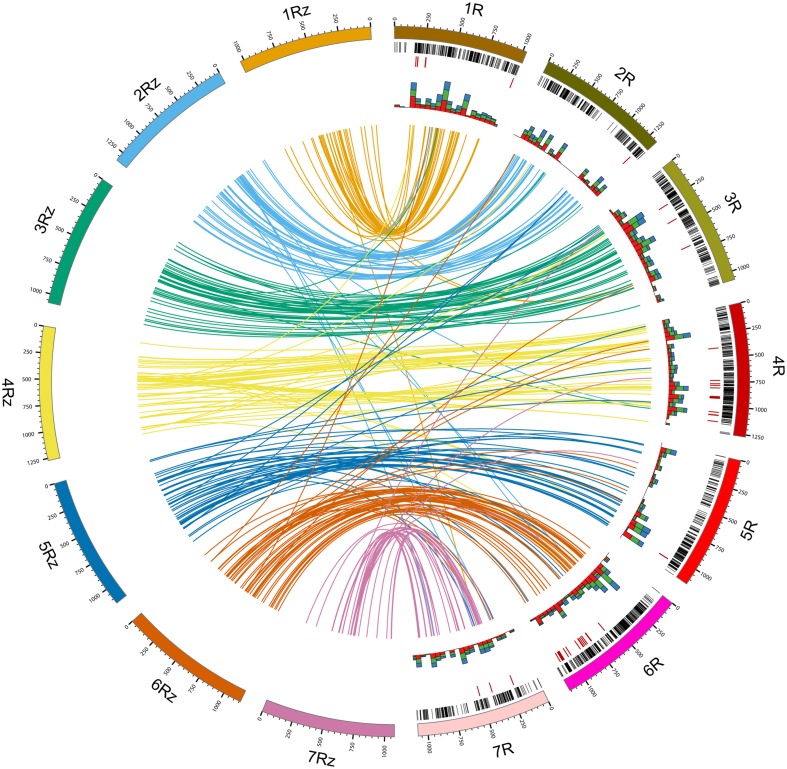
**DArT Sequence-based consensus maps comparison and functional annotation**. Lines inside the circle (colored according to rye genome zippers) connect positions of DArT sequences in rye consensus map (1R–7R; Milczarski et al., 2011) with best hit positions in rye genome zippers (1Rz–7Rz; Martis et al., 2013). Rye genetic map was scaled according to physical length of rye chromosomes. Black bands indicate distribution of DArT marker sequences along the genetic map. Red bands indicate positions of DArT marker sequences putatively associated with disease resistance. Stacked histograms (the innermost data track) indicate the total number of gene ontology terms belonging to the molecular function (red), biological process (green), and cellular component (blue) categories (summed over 500 Mbp intervals).

Following Blast2Go analysis we obtained significant hits for 1,516 unique DArT sequences (40.6% of the non-redundant sequences), with 319 hits to ‘hypothetical,’ ‘predicted,’ or ‘uncharacterized’ proteins. Of the remaining 1,198 sequences (32.06% of the non-redundant set), where a putative function could be attributed, 97 (2.6% of the non-redundant set) turned out to be associated with transposable elements (TEs). Only five of these sequences were identified previously as repeat-containing after the search against the TREP database. On the other hand, putative, TE unrelated functions could be attributed to 411 DArT sequences, which did not align to the rye sequence sets in homology analysis (see below for details), and to 139 sequences, which did not align to any of the analyzed sets. Putative functions associated with disease resistance were attributed to 132 sequences, including 65 DArT sequences which did not align to the rye sequence sets in the homology analysis. The highest number of DArT sequences with putative disease resistance-related functions was found in the chromosomes 4R and 6R (29 and 24 sequences, respectively, with the number of sequences on the remaining chromosomes ranging from 2 to 12). The numbers of disease resistance-related DArT sequences located in particular chromosomes showed strong positive correlation with the total numbers of non-redundant DArT sequences with defined chromosomal location (Spearman’s rank correlation, ρ = 0.90, *p*-value = 0.0056). However, the putatively disease resistance-related DArT sequences were not dispersed over the entire length of chromosomes 4R and 6R, but located in the long arms of both chromosomes (**Figure 3**).

Gene ontology terms were assigned to 1,234 sequences, one GO term was assigned to 274 sequences, to the remaining sequences two or more GO terms were assigned. The numbers of GO terms obtained for cellular components, biological processes, and molecular functions were 1,013, 1,903, and 1,724, respectively. Percentages of GO terms assigned to functional groups within cellular component, biological process, and molecular function categories are shown in **Supplementary Figure S1**. Enzyme codes were obtained for 210 sequences. Detailed information concerning functional annotation of DArT sequences can be found in the **Supplementary Table S2**.

### Homology Search within *Pooideae* Genomes and Rye Sequence Sets

**FIGURE 4:**
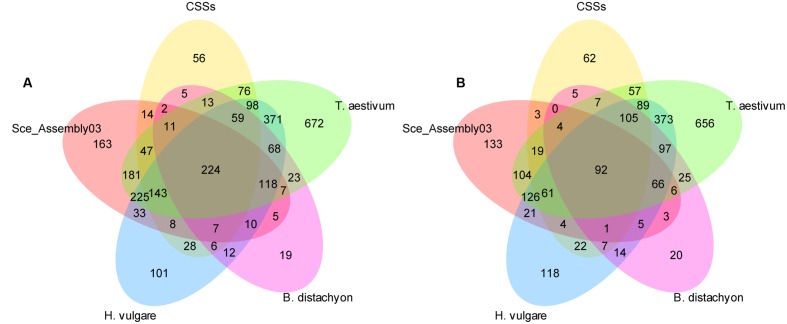
**Homology of the non-redundant rye DArT marker sequences to five *Pooideae* sequence sets**. Number of non-redundant rye DArT markers sequences aligning to the relevant sequence sets at two stringency settings at: **(A)** minimum identity length 100 bp, **(B)** minimum identity length 200 bp.

We performed two homology searches against *Pooideae* reference genomes and rye sequence sets. First we used the settings proposed by Martis et al. (2013), then we increased the minimum alignment length to 200 bp. The number of DArT sequences aligning to the respective sets at both stringency setting is shown in **Figure 4** and in **Table 1**. Since the increasement of the alignment length to 200 bp resulted in a lower number of sequences that aligned to multiple sequence sets, without drastic reduction in the number of sequences that aligned only to a single data set (**Figure 4**), and additionally limited the number of multiple hits for a given DArT marker within a single data set (this was particularly pronounced in the case of the wheat genome), we decided to use the results of the more stringent search in the subsequent analyses. Only for the EST-resource (Sce_Assembly03) the alignment length 100 bp was retained, to allow for detection of hits involving DArT sequences containing exon/intron junctions. Of the 3,737 unique DArT sequences 2,456 (65.7%) aligned to at least one sequence resource at the adopted settings. The numbers of DArT sequences aligning to reference genomes of *Brachypodium*, barley and wheat were 457, 1,201, and 1,887, respectively, while only 1,198 and 538 sequences, respectively, aligned to CSS and SceAssembly03 sequences. In total 2,337 DArT sequences didn’t align to the rye sequence sets included in the analysis (see **Supplementary Table S3** for details).

**Table 1:** Homology of the non-redundant rye DArT markers to five *Pooideae* sequence sets.

Sequence set	*E*-value	Minimum percent identity	Minimum alignment length	Number of BLASTN hits	Number of DArT sequences aligned
*Brachypodium distachyon* genome	10–5	75	100	851	589
			200	596	457
*Hordeum vulgare* genome	10–5	85	100	8825	1511
			200	2497	1201
*Triticum aestivum* sequence	10–5	90	100	126932	2336
			200	27509	1887
Sce_Assembly03	10–5	90	100	3518	1198
			200	967	648
Rye CSSs	10–5	90	100	2317	797
			200	1266	538

*Total number of non-redundant rye DArT markers sequences aligning to the relevant sequence sets at two stringency settings*.

**FIGURE 5:**
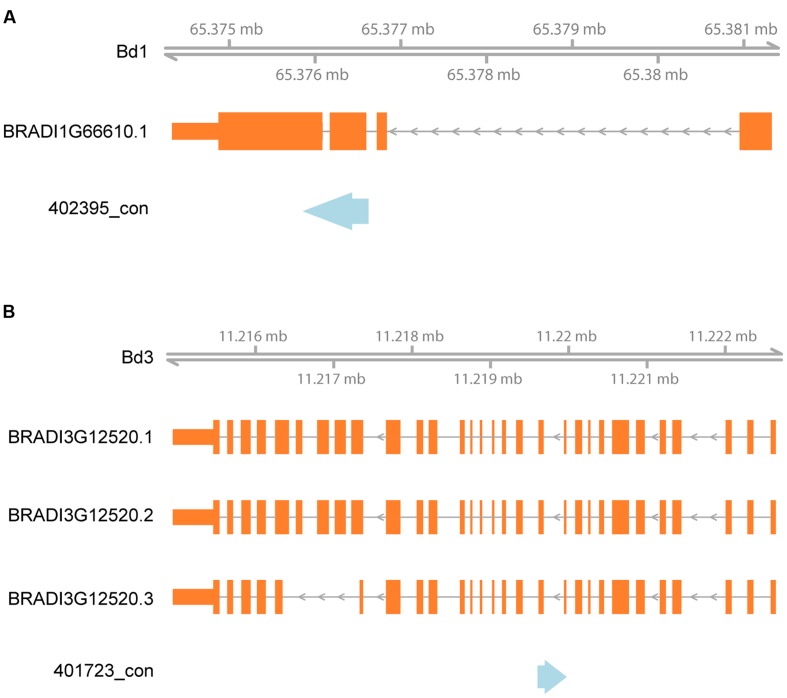
**Alignment of rye DArT markers sequences to *Brachypodium* in relation to gene models**. Blue arrows indicate rye DArT marker sequences, orange rectangles – gene models: **(A)** alignment of DArT marker sequence 402395_con, **(B)** alignment of DArT marker sequence 401723_con.

We additionally analyzed the distribution of the aligned DArT sequences relative to the position of annotated genes within the *Brachypodium* reference genome. Of 457 sequences, that gave a hit against the *Brachypodium* genome 430 (94.1%) were located in genes (minimum sequence overlap of 50 bp, **Supplementary Table S2**). Two examples of alignment of rye DArT markers sequences in relation to *Brachypodium* gene models are shown in **Figure 5**.

### Integration of Unique Rye DArT Marker Sequences into Rye Genome Zippers and Map Comparison

Information concerning genetic map position was available for 2383 unique DArT marker sequences and it corresponded to 1887 map locations. In total 1534 markers had unique map locations, the remaining 849 markers were mapped to 353 locations. The number of markers sharing the same map location ranged from two to nine. Distribution of the unique DArT sequences along the rye genetic map is shown in **Figure 3**. Information concerning genetic map locations of the unique DArT marker sequences is given in the **Supplementary Table S2**. The Spearman’s rank correlation test revealed a strong positive correlation (ρ = 0.89, *p*-value = 0.0123) of the number of unique map location of DArT markers sequences in the individual chromosomes with the numbers of DArT markers mapped in the individual chromosomes in the consensus map (Milczarski et al., 2011). Despite the overall good genome coverage, there are few chromosomal regions in the map were the marker sequences are missing, the largest region lacking sequenced DArT markers is located in the proximal part of the 2RL chromosome.

Based on the best BLAST hits against Sce_Assembly03 and rye CSSs 515 unique DArT sequences could be incorporated into rye genome zippers (Martis et al., 2013). Information concerning the position of DArT sequences in the rye genome zippers is given in the **Supplementary Table S2**. Genetic map locations were available for 357 (69.3%) of these DArT sequences (from 33 for 7R to 60 for 6R). In total 85.4% of DArT sequences were incorporated into rye genome zippers at positions on corresponding chromosomes with respect to location of DArT marker sequences on the DArT-based consensus map. The highest discrepancy with respect to chromosomal location was observed for chromosomes 7R and 6R – respectively, 25.6 and 17.5% of DArT markers were incorporated into genome zippers of different chromosomes. The highest congruency was observed for chromosome 1R – 93.5% of DArT sequences mapped *in silico* on the genome zipper of chromosome 1R. Moreover, as shown in **Figure 3** some differences with respect to location within individual chromosomes were also apparent. An inverted order of DArT sequences in the long arm of the chromosome 4R is especially noticeable. On the other hand a relatively consistent marker order was apparent in 4RS, 3R, and 6R.

### Development of SSR Markers

Since rye transcriptome sequences (Haseneyer et al., 2011) had already been used for the development of SSR markers we used for this purpose only these 2539 unique DArT sequences for which no hits against the Sce_Assembly 03 were obtained. We identified 238 SSR motifs, the majority of them (117 motifs) were mononucleotide repeats, followed by dinucleotides (57 motifs), trinucleotides (27 motifs), tetranucleotides (nine motifs), and penta- and hexanucleotides (one motif each). The remaining 26 motifs designated by the SSR detecting algorithm as compound repeats were basically two stretches of microsatellite repeats separated by a non-SSR- or unknown sequence (Ns). Primer design was possible for 195 newly identified motifs. A list of the identified SSRs and sequences of primers are given in the **Supplementary Table S4**. For the experimental validation, we have chosen motifs with the basic repeat unit length >1 and the total repeat length ≥15 bp (56 primer pairs). Amplicons of expected size were obtained for 48 primer pairs. Polymorphic and easy to score products were obtained using 20 (35.7%) of the tested primer pairs (**Table 2**). The number of detected alleles ranged from two to seven (average 4.65), while the PIC values ranged from 0.31 to 0.82 (average 0.66).

**Table 2:** Characteristics of SSR markers developed from rye DArT marker sequences.

SSR marker name	DArT sequence ID	Chromosomal location^a^	Number of allels	Expected amplicon size [bp]	Allele size range [bp]	PIC value
SSR_400236_con	400236_con	1R	6	216	208-230	0.59
SSR_402546_con	402546_con	1R	6	161	155-184	0.73
SSR_402642_con	402642_con	1R	2	205	200-206	0.45
SSR_402421	402421	2R	7	249	215-250	0.65
SSR_508543_con	508543_con	2R	7	158	155-173	0.82
SSR_508929_1	508929	3R	3	203	190-212	0.74
SSR_508929_2	508929	3R	4	165	156-176	0.79
SSR_399980	399980	3R	8	131	121-139	0.79
SSR_509517	509517	4R	5	256	210-235	0.31
SSR_508692_con	508692_con	5R	6	215	198-218	0.72
SSR_505581	505581	5R	4	197	182-208	0.81
SSR_400330_con	400330_con	5R	2	168	161-170	0.79
SSR_401898_con	401898_con	6R	2	219	210-220	0.75
SSR_505949_con	505949_con	6R	4	164	157-171	0.73
SSR_508976	508976	7R	4	199	190-210	0.50
SSR_402502	402502	n.a.	7	185	173-192	0.64
SSR_398598	398598	n.a.	5	160	157-176	0.59
SSR_508416	508416	n.a.	4	222	203-224	0.81
SSR_390082	390082	n.a.	3	141	138-142	0.48
SSR_507134	507134	n.a.	4	138	134-148	0.54

*^a^Chromosomal location of the DArT sequence from which the relevant SSR marker was developed*.

## Discussion

There are several strategies that can be used in genomics research to circumvent problems imposed by large genome size and complexity. These strategies usually rely on addressing the gene space, but many of them, such as RNA-Seq (Wang et al., 2010) or exome capture (Warr et al., 2015), are beyond reach of scientists working on crops of lesser economical importance, due to high cost involved or because an extensive preexisting genome sequence knowledge is required. Diversity Arrays Technology (Jaccoud et al., 2001) is an affordable, sequence independent alternative, which delivers several thousand genetic markers in a single assay. The preferential sampling of gene-rich regions is achieved through the use of methylation sensitive restriction enzymes in the genome complexity reduction stage, which is a crucial element of the DArT protocol. DArT genotyping panels had been established for a considerable number of species, including orphan/resource poor crops (Gupta et al., 2008; Petroli et al., 2012 for a reference list). To date sequence information was obtained for sets of selected DArT markers from a few genotyping panels. The species in question include *Eucalyptus* (Petroli et al., 2012), oats (Tinker et al., 2009), sugarcane (Aitken et al., 2014), wheat (Marone et al., 2012), apple (Schouten et al., 2012), and potato (Traini et al., 2013). This is the first report concerning rye DArT marker sequences. At the same time rye has the largest monoploid genome size of all species for which DArT marker sequences were obtained and analyzed, and it could serve as a model for verification of the DArT markers’ ability to efficiently target low copy sequences in a large and highly repetitive genome.

### Sequence Analysis of the Rye DArT Markers

The set of 6,177 DArT sequences described in our study is one of the largest analyzed so far, alongside *Eucalyptus* (Petroli et al., 2012) and sugarcane (Aitken et al., 2014) data sets, which consisted of 6,918 and 7,846 DArT marker sequences, respectively. The average sequence length of a rye DArT marker (508 bp) is within range of those reported for other species: Sugarcane – 465 bp (Aitken et al., 2014), oats – 496 bp (Tinker et al., 2009), *Eucalyptus* – 534 bp (Petroli et al., 2012) and similarly like in other studies, in which such data was reported (Tinker et al., 2009; Schouten et al., 2012), the majority of contigs obtained after redundancy analyzes consisted of two or three sequences. On the other hand, the sequence redundancy (39.5%) was generally slightly higher than in other species – the redundancy estimates were 31.22%, 32.73% and between 33.75 and 44.14% for sugarcane, oats, and *Eucalyptus*, respectively (Tinker et al., 2009; Petroli et al., 2012; Aitken et al., 2014). Much smaller DArT sequence redundancy estimates were also reported: 15.6% for 384 apple DArT markers (Schouten et al., 2012), and 12.5% for 2,000 wheat DArT markers (Marone et al., 2012). Those small values, however, are very likely the result of a strong preselection of DArT clones prior to sequencing, based on marker scores within mapping progenies and/or diverse accessions. As observed by Petroli et al. (2012) the observed redundancy level is influenced by several factors, such as the structure of the particular genome, the diversity of accessions used during the creation of the genotyping panel, and the number of selected clones. In our opinion, there are two major reasons for the relatively high sequence redundancy of the rye DArT markers. Firstly, with its 1520 clones the rye genotyping array is one of the largest operational DArT arrays, and, apart from 768 clones rearrayed from the wheat genotyping array, the majority of the clones included in the array were not subjected to prescreening with respect to polymorphism detection efficiency nor the distinctness of observed segregation patterns (Bolibok-Bragoszewska et al., 2009). Secondly, for the sequencing we have chosen all the DArT markers, which segregated in at least one mapping population, even if multiple markers mapped to the same map position.

Based on a homology search within the TREP database only 1.26% of non-redundant DArT sequences were found to contain fragments of repeat elements, with Gypsy LTR retrotransposons and Mariner DNA transposons being the most abundant classes (48.2 and 35.1% of hits, respectively), followed by Copia LTR retrotransposons (4.6%). This is only in partial agreement with the previous results concerning the rye repeat elements. Analyzes of sequence reads from the flow sorted 1RS chromosomes (Fluch et al., 2012) revealed, that Gypsy LTR retrotransposons were the most frequent TEs (62.36% of known repeat elements identified in the study), followed by Copia LTR retrotransposons (10.61%) and CACTA DNA transposons (9.24%), whereas the frequency of Mariner DNA transposons was only 0.28%. It was observed in wheat (The International Wheat Genome Sequencing Consortium (IWGSC), 2014) that there is a clear reduction and also a change in a relative frequencies of various TEs in the vicinity of genes. Since DArT markers are associated with the gene-rich regions (which will be discussed in more detail later on) this is probably the reason for the discrepancy between frequency levels of different TE-types observed in rye DArT marker sequences and in sequence reads from the 1RS chromosome.

### DArT Technology Effectively Targets Low Copy/Genic Sequences in the Large and Repetitive Rye Genome

Previous analyses of DArT sequences in other species indicated that DArT markers tend to be located in gene-rich regions. In oats ca. 50% of non-redundant DArT sequences exhibited homology to known or predicted gene sequences, however, a detailed functional annotation was not performed (Tinker et al., 2009). Similarly, 64% of wheat DArT clones were found to represent expressed sequences, putative non-TE-related functions were attributed to 593 sequences (33.8% of the total set; Marone et al., 2012). In sugarcane 50.2% of DArT sequences exhibiting homology to the sorghum genome sequence aligned to annotated genes (Aitken et al., 2014). Very striking results were obtained in *Eucalyptus* (Petroli et al., 2012), where due to the availability of the reference genome sequence it was possible to directly asses the distribution of DArT sequences relative to gene models. It was found that close to 70% of DArT marker sequences aligned at 0 bp from the closest gene model.

The combined results of functional annotation and of the search against the TREP database indicate that only 170 rye DArT marker sequences (4.5%) contain known repetitive elements, while putative, non-TE-related functions could be attributed to 29.4% of the non-redundant DArT marker sequences. Furthermore 94.1% of DArT sequences, exhibiting similarity to the *Brachypodium* genome aligned within annotated genes. It should be, however, noted that the preferential selection of mapped DArT markers for sequencing (segregating in the mapping progenies in Mendelian fashion) likely introduced a certain bias into the analyzed set and the frequency of repetitive and gene-related sequences in the unsequenced fraction (ca. 46.4%) of the rye genotyping array is probably different. Nevertheless our results demonstrate clearly, that DArT markers effectively target the gene space even in the very large, complex, and highly repetitive rye genome.

### Homology Search within *Pooideae* Sequence Resources and Functional Annotation

The numbers of sequences that aligned to the reference genomes of *Brachypodium*, barley and wheat are in accordance with the phylogenetic relationships between these species. The relatively small number of sequences that aligned to the CSS set could seem surprising, it is, however, consistent with the low coverage of the CSS – average sequence coverage of 1.04-fold, average expected base pair coverage of 64.6%, and average marker detection rate (which was estimated by comparing the CSS reads against genetically anchored sequence markers) of 78.7% (Martis et al., 2013). The percentage of DArT sequences, which gave no hits to any of the reference *Pooideae* genomes (43.6%) is higher than that observed previously in similar analyses involving Sce_Assembly03 (31%) (Haseneyer et al., 2011). The existence of species and tribe specific genes and gene families was proposed as the reason for this relatively high proportion of transcript assemblies, which did not exhibit significant similarity to any public *Poaceae* sequences (Haseneyer et al., 2011). Additional probable factors that contributed to the higher value observed in our study are: a narrower set of sequence resources involved in the homology searches – in case of Sce_Assembly03, apart from the *Brachypodium* genome, wheat flcDNAs and barley ESTs, also maize, rice, and sorghum reference genomes were used (Haseneyer et al., 2011), and more stringent search settings. On the other hand, for a part of DArT sequences we observed multiple hits in the respective data sets. This probably reflects the ancient whole genome duplication event, which predates the divergence of grass genomes (Yu et al., 2005) and/or the characteristic for the *Triticeae* genomes abundance of pseudogenes and gene fragments (Wicker et al., 2011).

The homology search within the five *Pooideae* sequence sets, coupled with the results of functional annotation revealed that the rye DArT clones characterized in this study contain novel, meaningful sequence information, since putative, TE-unrelated functions could be attributed to 411 DArT sequences, which gave no hits to the rye sequence sets in homology analysis, and to 139 sequences, which gave no hits to any of the analyzed sets. Disease resistance-related annotations were obtained for 65 and 32 of these sequences, respectively. They could be thus of direct benefit to researchers and breeders. The putatively disease resistance-related sequences were preferentially located in the long arms of chromosomes 4R and 6R. Interestingly, both these chromosomes were indicated as containing regions targeted by selection in current breeding programs (4R) and during domestication (6R; Bolibok-Bragoszewska et al., 2014). While disease resistance is one of the major objectives in cereal breeding, it is not one of the primary traits typically associated with domestication (Fuller, 2007; Purugganan and Fuller, 2009). Further research is needed to determine if the locations of putatively disease resistance-related sequences and locations of genome regions subjected to selection pressure during domestication and breeding coincide.

### Integration into Rye Genome Zippers and Comparison of Consensus Maps

Integration of 515 DArT sequences into rye genome zippers provides a starting point for the integration of DArT and SNP-based genomics resources, simplifies access to the rye sequence sets and conserved synteny information contained in genome zippers, and we believe that it will contribute to a better exploitation of these resources in research and breeding. It also enabled a first comparison of the DArT- and the transcript-based consensus maps (Milczarski et al., 2011; Martis et al., 2013).

For the DArT sequence-based consensus map comparison we used the locations of the corresponding DArT markers in the rye consensus map (Milczarski et al., 2011). This information was available for 2383 DArT sequences and it corresponded to 1887 map locations. There were thus 353 cases where at least two DArT markers with unique sequences shared the same genetic map location. Similarly in oats (Tinker et al., 2009) identical segregation patters in mapping progenies were observed for some DArT markers with unique sequences. As suggested by Tinker at al. (2009) such DArT markers represent probably distinct genomic loci, which could be not resolved because they are in linkage disequilibrium or the size of the mapping population was not sufficient for a crossing-over event to occur between those loci. The analysis of distribution of sequenced DArT markers across the genetic map revealed an overall good map coverage but also lack of available sequences in few short chromosomal segments, therefore, a targeted sequencing of additional DArT markers from the particular regions would be advisable.

In result of consensus maps comparison certain inconsistencies with respect to chromosomal location of markers and marker orders were revealed. Marker order inconsistencies are not a rare occurrence in consensus mapping (Mace et al., 2008; Milczarski et al., 2011) and are observed even between different map versions of the same population (Petroli et al., 2012). Several sources of experimental error, including missing values, genotyping errors, and segregation distortion are known to influence the estimations of marker order (Hackett and Broadfoot, 2003). It was also demonstrated that limited progeny size (below 200 individuals) can compromise the accuracy of genetic maps and result in inverted marker orders (Ferreira et al., 2006). The average number of individual per mapping population was 123 and 112 in the SNP and DArT-based studies, respectively, with the minimum population sizes of, respectively, 69 and 82. At present, an existence of chromosomal rearrangements between the parental lines also cannot be excluded. Although the map comparison is based solely on sequence homology and the experimental validation involving DArT or SNP genotyping of the relevant progenies has been not attempted, it would be advisable to resolve the issue of map inconsistencies before using either genetic map for anchoring of sequence scaffolds during physical map construction.

### Development of SSR Markers Based on DArT Sequences

SSRs are easy to use, robust, and powerful markers and despite the establishment of highly parallel SNP (Gupta et al., 2008) or next-generation-sequencing (Davey et al., 2011) genotyping technologies they are still widely used in germplasm characterization (Laidò et al., 2013; Diez et al., 2015) and linkage mapping (Petroli et al., 2012; Martis et al., 2013). Of the many available methods of SSR marker development, to date, genomic libraries (Saal and Wricke, 1999; Bolibok et al., 2006), BAC-end-sequences (Kopecký et al., 2009), and EST sequences (Hackauf and Wehling, 2002; Haseneyer et al., 2011) had been used for this purpose in rye. To our knowledge a development of SSR markers based on DArT marker sequences has not been attempted before in any species. The percentage of primer pairs producing polymorphic product was lower but comparable to those obtained for EST derived SSR markers [47.2% (Hackauf and Wehling, 2002) and 39.4% (Martis et al., 2013)] but markedly higher than in the case of SSRs from genomic libraries [12% (Saal and Wricke, 1999)]. The average PIC value and the average number of alleles obtained in our study are comparable with those observed in a recent assessment of genetic diversity and population structure of 367 rye accessions by Targońska et al. (2016), who used a set of 22 preselected, genomic library- and EST-derived SRRs – 0.66 and 4.65, respectively. We present here only the results of a preliminary experimental validation of the newly developed markers, and their suitability for large germplasm characterization studies is to be determined. Nevertheless we expect that these markers will be helpful during construction of genetics maps. While no substantial increase in the number of rye SSRs was achieved with the newly developed markers, we have demonstrated that DArT sequences can be a source of SSR markers. The opportunity of SSR marker development could be a considerable additional benefit of DArT marker sequencing in orphan crops/genomics-resources-poor species.

## Conclusion

Our data demonstrate that DArT technology effectively targets low copy/genic sequences in the very large, complex, and repetitive genome of rye and delivers markers with associations to traits relevant in breeding, such as disease resistance. Through the annotation of putative gene functions and the alignment of DArT sequences relative to reference genomes we obtained information, that will complement the results of the studies, where DArT genotyping was deployed by simplifying the GO and microcolinearity based identification of candidate genes. We also demonstrated for the first time that sequencing of DArT markers provides an additional benefit relying in the possibility of SSR marker development. Through the use of the newly obtained DArT marker sequence data we were able to reveal obvious inconsistencies between two rye consensus maps and thus identify an important issue for further research.

## Author Contributions

Conceived and designed the study: HB-B. Contributed reagents/materials/analysis tools: PG, MP, GU, MT, MW, AK, HB-B. Performed the experiments: KT and SS. Analyzed the data: PG, MP, GU, KT, SS, SA, AK, and HB-B. Wrote the manuscript: HB-B.

## Conflict of Interest Statement

AK and GU are employees of Diversity Arrays Technology Pty Ltd, which offers genome profiling services and where the microarray, whose genomic characterization is described in this report was developed. This fact, however, has not interfered whatsoever with the full, objective, transparent, and unbiased presentation of the research results described in the manuscript nor alters the authors’ adherence to all the Frontiers in Plant Science policies on sharing data and materials. All the other authors declare that the research was conducted in the absence of any commercial or financial relationships that could be construed as a potential conflict of interest.

## Footnotes

1 http://www.diversityarrays.com/dart-map-sequences

2 http://wheat.pw.usda.gov/ITMI/Repeats/

3 http://plants.ensembl.org/index.html

## Supplementary Material

FIGURE S1 **Percentages of GO terms assigned to functional groups**. Percentages of GO terms assigned to functional groups within **(A)** biological process, **(B)** cellular component and **(C)** molecular function categories.

TABLE S1 **List of DArT sequence IDs**.

TABLE S2 **Functional annotations of non-redundant rye DArT marker sequences**.

TABLE S3 **Homology search results for 3737 non-redundant rye DArT marker sequences**.

TABLE S4 **SSR motifs and primers for their amplification**.
