# Supplementary figures and images for: DArT Markers Effectively Target Gene Space in the Rye Genome
Source: Front Plant Sci. 2016 Oct 26;7:1600. doi: 10.3389/fpls.2016.01600 (PMC5080361; doi:10.3389/fpls.2016.01600)

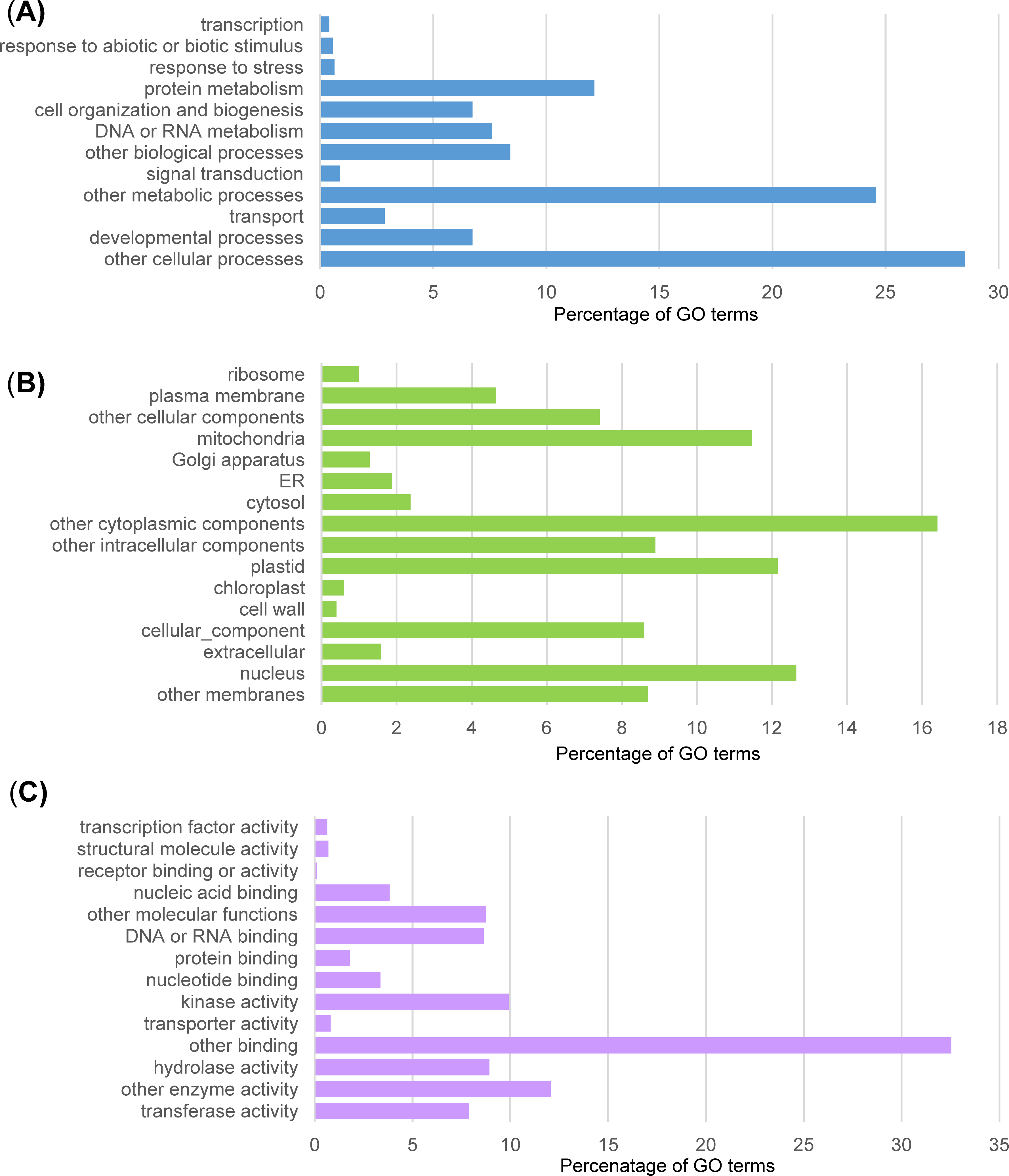

Supplement: FIGURE S1 — Percentages of GO terms assigned to functional groups. Percentages of GO terms assigned to functional groups within (A) biological process, (B) cellular component and (C) molecular function categories. [file Image_1.TIF]
